# Accuracy of Caregiver Dosing of Acetaminophen and Ibuprofen for Febrile Children Presenting to a Saudi Arabian Tertiary Hospital Emergency Department

**DOI:** 10.3390/healthcare14193308

**Published:** 2026-10-04

**Authors:** Youssef A. Alqahtani, Ayed A. Shati, Saleh M. Al-Qahtani, Syed Esam Mahmood, Ausaf Ahmad

**Affiliations:** 1 Department of Child Health, College of Medicine, King Khalid University, Abha 62529, Saudi Arabia; yal-qahtani@kku.edu.sa (Y.A.A.); ashati@kku.edu.sa (A.A.S.); smuadi@kku.edu.sa (S.M.A.-Q.); 2 Department of Family and Community Medicine, College of Medicine, King Khalid University, Abha 62529, Saudi Arabia; 3 Department of Community Medicine, Kalyan Singh Government Medical College, Bulandshahr 203001, Uttar Pradesh, India; ausafahmad86@gmail.com

**Keywords:** acetaminophen, ibuprofen, caregivers, children, correct dose, factors associated, practice, Saudi Arabia

## Abstract

**Background:** Fever and its related symptoms are common reasons for pediatric emergency department (ED) visits. Parents are often concerned about fever in children, and a frequent reason for ED visits is the persistence of fever despite the administration of antipyretics at home. It is hypothesized that caregivers often administer incorrect doses of acetaminophen and ibuprofen. Objective: To assess caregivers’ dosing practices in children with fever by evaluating the accuracy of acetaminophen and ibuprofen dosing and to identify factors associated with inaccurate dosing in Saudi Arabia. **Methodology:** A cross-sectional survey was conducted over a six-month period from 1 January 2022 to 30 June 2022 in the emergency department. We included caregivers of children under 14 who received a quantifiable dose of acetaminophen or ibuprofen for fever. Data were collected using a structured questionnaire. Multivariable regression analysis was utilized to identify factors associated with inaccurate dosing. **Results**: A total of 330 caregivers of children were included in the study. The caregivers’ ages ranged from 18 to 60 years, with a mean age of 32.3 ± 6.4. Of these, 148 (44.8%) had university-level education or higher, while 33 (10%) had education below high school level. Only 81 caregivers (24.5%) administered the correct dose of medication. In contrast, 118 (35.8%) gave subtherapeutic doses, 40 (12.1%) administered higher-than-recommended doses, and 91 (27.6%) were unsure about the correct dose. Multivariable regression indicated that caregivers with education below high school (aOR = 2.65; 95% CI: 1.30–5.40 for low dose) and those making self-directed dosing decisions (aOR = 2.10; 95% CI: 1.15–3.85 for low dose) were significantly associated with incorrect dosing. **Conclusions:** The study indicates that approximately three-quarters of caregivers either administered incorrect doses of acetaminophen or ibuprofen or were unsure of the correct dose. Given that 93% of caregivers were unaware of medication concentrations, targeted educational interventions are required to improve dosing accuracy.

## 1. Introduction

Fever is a common and usually benign symptom in children, but its management is crucial for ensuring child safety [1,2]. Effective antipyretic treatment is essential in preventing simple febrile seizures [3] as well as in managing children with cardiopulmonary conditions and increased intracranial pressure, where high body temperature can exacerbate symptoms [2,4].

Acetaminophen and ibuprofen are the most frequently used antipyretic medications in children [5]. This excerpt from the *Nelson Textbook of Pediatrics* warns that repeated high doses of acetaminophen (e.g., 90 mg/kg/day) in children can deplete liver glutathione, risking acute liver injury, while inappropriate ibuprofen dosing carries distinct renal and gastrointestinal toxicity risks [6].

Several studies have indicated that caregivers may unintentionally administer incorrect doses of acetaminophen, often slightly exceeding the recommended amount, which can increase the risk of adverse effects [7,8,9,10,11]. In Saudi Arabia, there is evidence that parental awareness regarding childhood fever management is limited and frequently marred by misconceptions. Factors such as maternal education, age, employment status, and family size are significantly associated with caregivers’ knowledge and beliefs [12]. Additionally, research from Makkah identified notable gaps in caregivers’ understanding of pediatric fever treatment with acetaminophen and ibuprofen, with 47.1% expressing concern about potential acetaminophen-induced liver damage [13]. A study conducted in southwestern Saudi Arabia further indicated that caregivers’ education level significantly impacted treatment duration, dosage, and administration frequency of acetaminophen, with all findings reaching statistical significance [14].

The lack of accurate dosing information and the potential for incorrect administration of antipyretic medications highlight the need for this study. By investigating caregivers’ medication-dosing practices for febrile children, we aimed to identify factors associated with dosing inaccuracies and inform strategies to promote the safe use of acetaminophen and ibuprofen in pediatric populations.

## 2. Methodology

A cross-sectional study was conducted over a six-month period from 1 January 2022 to 30 June 2022 in the emergency department (ED) at the King Faisal Specialist Hospital after obtaining ethical approval from the Research Ethics Committee of King Khalid University (ECM#2022-4798). Participants were recruited using consecutive convenience sampling during randomized shifts to minimize selection bias while accommodating the unpredictable workflow of the ED.

Data collection shifts were assigned using a computerized random number generator to distribute coverage evenly across various times and days.

The sample size was determined using the standard Cochrane formula for cross-sectional studies (n = Z^2^ P(1 − P)/d^2^). Assuming a conservative 50% prevalence of incorrect dosing to yield the maximum required variance, a 95% confidence level (Z = 1.96), and a 5.5% margin of error, the minimum required sample size was calculated to be 317 [15]. A total of 346 cases were screened. We excluded 10 for unquantifiable doses and six for lack of caregiver presence, successfully recruiting a final sample of 330 caregivers.

All caregivers of children younger than 14 years who had received a quantifiable dose of acetaminophen or ibuprofen within 24 h before the ED visit were eligible for inclusion. A quantifiable dose was defined as an exact amount in milliliters or milligrams or a standard suppository unit. The medication must have been given for fever, defined as a temperature of 38 °C (100.4 °F) or higher. Treatment was considered to have been initiated when the child’s temperature reached or exceeded this threshold, as measured at home. Parents were also asked what they considered to constitute a fever. Children who received fever medication other than the study drugs, were given an unknown dose or received the drug for a reason other than fever, or were not accompanied by a caregiver were excluded. The study was approved by the Research Ethics Committee, King Khalid University (protocol code ECM#2022-4798, 1 January 2022). The committee approved obtaining verbal informed consent from caregivers before the interviews; therefore, a waiver of written consent was granted. Data were collected from caregivers using a pre-structured standardized questionnaire agreed upon by the research committee that was developed by researchers after an intensive literature review of similar articles [12,16,17,18] and expert consultation. The questionnaire was paper based and interviewer administered. The questionnaire was pilot tested with 30 participants, leading to improvements in clarity and structure. It demonstrated high reliability with a Cronbach’s alpha of 0.85. To ensure cultural relevance, it was accurately translated from English to Arabic through a careful, two-step process involving back-translation, ensuring consistent interpretation across language groups. The questionnaire was completed first by the principal investigators during their randomized shifts. Data collected included demographic data for caregivers and their children, type, dose, route of administration, and frequencies of acetaminophen/ibuprofen drugs. Children’s weight was measured in the emergency department.

### 2.1. Definition of Correct and Incorrect Dosing

Dosing accuracy was evaluated strictly based on the child’s weight (mg/kg) at the time of presentation, utilizing *The Harriet Lane Handbook* as the primary reference guideline. For acetaminophen, a correct dose was defined as 10 to 15 mg/kg per dose. For ibuprofen, a correct dose was defined as 5 to 10 mg/kg per dose. Doses administered below these specific therapeutic thresholds were categorized as “underdosing” (subtherapeutic), while doses exceeding these limits were categorized as “overdosing” (supratherapeutic). Caregivers who responded “don’t know” regarding the exact dose administered were categorized as indicating inaccurate dosing practice, as this uncertainty inherently precludes accurate weight-based dosing.

### 2.2. Data Analysis

After data were extracted, they were revised, coded, and fed to statistical software IBM SPSS version 22 (SPSS, Inc. Chicago, IL, USA). Descriptive analysis based on frequency and percent distribution was done for all variables, including caregiver and child demographic data, drug administration parameters, and caregiver practices. Cross-tabulation and bivariate analysis (Pearson Chi-Square and Fisher’s Exact tests) were utilized to examine baseline associations. Following this, a multivariable multinomial logistic regression analysis was conducted to simultaneously control for confounders and identify independent factors associated with inaccurate caregiver dosing. The outcome variable consisted of three inaccurate dosing categories (low dose, high dose, don’t know), with “correct dose” set as the standard reference category. Results for the multivariable analysis are presented as adjusted odds ratios (aORs) with 95% confidence intervals (CIs). To account for multiple comparisons and reduce the risk of Type I errors, *p*-values were adjusted using the false discovery rate (FDR) method. A two-tailed *p*-value less than 0.05 was considered statistically significant. Caregivers who responded “don’t know” regarding the administered dose were retained as a distinct category reflecting inaccurate dosing practice, while true missing data were handled using listwise deletion.

## 3. Results

The caregivers of a total of 330 children aged between 0 and 7 years (168 males and 162 females) were included in the study. Respondents’ ages ranged from 18 to 60 years with a mean age of 32.3 ± 6.4 years old. The mean weight of the pediatric patients was 13.8 ± 5.2 kg. Stratified by age, the mean weights were 7.5 ± 1.8 kg for <1 year, 11.2 ± 2.1 kg for 1–2 years, 14.6 ± 2.8 kg for 2–4 years, and 20.4 ± 3.5 kg for 5–7 years. A total of 151 (45.8%) respondents were the children’s mothers, and 81 (24.5%) were their fathers. In total, 86 (26.1%) were both parents, while 12 (3.6%) were relatives. A total of 129 (39.1%) caregivers were aged less than 30 years, while 36 were aged more than 40 years (10.9%). As for education, 148 (44.8%) caregivers had a university level of education, or above while 33 (10%) had a below high school level of education. A total of 143 (43.3%) respondents had one child aged below 7 years, and 168 (50.9%) had 2–3 children. Total of 102 (30.9%) caregivers had children aged 1–2 years, and 49 (14.8%) had children aged less than 1 year. In addition, 95 (28.8%) had children aged 5–7 years. A total of 168 (50.9%) caregivers’ children were males (Table 1).

Table 2 outlines the drugs given and their routes of administration. Fevadol and Adol were the most frequently administered medications, with syrup being the predominant formulation used by caregivers.

Table 3 summarizes caregivers’ dosing practices and dosing frequency. Most caregivers were unaware of the medication concentration (93.0%) or suppository strength (88.5%). Among those administering syrup, 46.7% reported giving less than 5 mL, while 43.6% gave 5–9 mL. The most commonly reported dosing interval was every 6 h (68.5%), and nearly half of caregivers (47.6%) relied on a doctor’s recommendation for the dose.

Table 4 summarizes caregivers’ practice regarding giving drugs and dose determinants. A total of 16 (4.8%) caregivers reported that they use acetaminophen with ibuprofen or diclofenac at the same time, 23 (7%) give the drug using more than one route at the same time, and 228 (69.1%) give treatment before attending the ER. A total of 45 (13.6%) of the caregivers reported weight as one of the drug dose determinants, and 42 (12.7%) reported age. However, 24 (7.3%) were unaware of dose determinants.

A total of 81 (24.5%) caregivers reported giving accurate drug doses. In contrast, 118 (35.8%) gave low doses, 40 (12.1%) gave high doses, and 91 (27.6%) were uncertain about the correct dose.

Table 5 examines baseline associations between caregiver characteristics and dosing accuracy (correct, low, high, or don’t know). No statistically significant overall associations were found for caregiver relationship (*p* = 0.814), age (*p* = 0.247), number of children (*p* = 0.285), education level (*p* = 0.201), frequency of drug intake (*p* = 0.412), or source of the dose recommendation (*p* = 0.165).

Table 6 presents the results of a multivariable multinomial logistic regression. It compares the odds of three distinct inaccurate dosing outcomes—low dose, high dose, and don’t know—against the baseline reference category of administering the correct dose. Caregivers with less than a high school education were significantly more likely to make errors. Compared to high school graduates, they had 2.65 times the odds of giving a subtherapeutic (low) dose and 3.10 times the odds of not knowing the correct dose at all. Conversely, higher education acted as a protective factor. Caregivers with a university degree or higher had significantly reduced odds of administering a low dose (aOR = 0.55) or being unaware of the dose (aOR = 0.45) compared to the high school reference group. When caregivers independently decided the dose themselves rather than following a doctor’s recommendation, they were at a significantly higher risk for dosing error. There was no statistically significant difference in dosing accuracy between following a doctor’s advice versus a pharmacist’s advice. Compared to caregivers aged 30–40, those over 40 were at a significantly higher risk for incorrect doses. They had more than double the odds of administering a low dose (aOR = 2.15) or responding “don’t know” (aOR = 2.45). Being under 30 did not show any statistically significant difference in dosing accuracy when compared to the 30–40 reference group. Whether the respondent was the mother, father, both parents together, or a relative made no significant difference in the accuracy of the dosing.

## 4. Discussion

Ibuprofen and acetaminophen are the most extensively used and accessible over-the-counter medications for management of fever and pain in children. Morton SM et al. [19] conducted a prospective study on children followed from birth and found that about 95% of children are exposed to acetaminophen by age 9 months. In spite of the frequent use of these medications, treatment recommendations for young children remain contradictory, particularly for infants during the first 6 months [20]. Irrespective of the drug use since early life, the maximum daily dose beyond the neonatal period differs from 60 mg/kg/d to 90 mg/kg/d [21,22]. Recommendations for ibuprofen use for the treatment of fever and pain in young children are considerably more variable. For example, the *New Zealand Formulary for Children*, which references the *British National Formulary for Children*, recommends ibuprofen use from age 1 month at 5 mg/kg/dose, 3 to 4 times daily, to a maximum dose of 30 mg/kg/d. On the other hand, in the USA, ibuprofen administration should only be given for children aged at least 6 months, with a higher maximum daily dose of 40 mg/kg/d [23].

The current study’s objective was to assess caregivers’ dosing practice of giving children with fever an accurate dose of acetaminophen or ibuprofen and to assess factors associated with inaccurate dosing in Saudi Arabia. The study showed that nearly one-fourth of the caregivers (24.5%) gave accurate drug doses. In contrast, low doses were given by more than one-third of them, and 12.1% gave high doses. Moreover, the other one-fourth (27.6%) were uncertain about the correct dose. It is clinically imperative to differentiate between these types of errors. Underdosing (subtherapeutic administration) primarily leads to unresolved fever, persistent parental anxiety, and unnecessary return visits to the emergency department. Conversely, overdosing (supratherapeutic administration) carries a severe, direct risk of cumulative hepatotoxicity for acetaminophen or renal and gastrointestinal toxicity for ibuprofen. As for factors associated with incorrect dosing, the study revealed that caregivers’ demographics had a significant relation with caregivers’ dosing practice. Significant factors were identified. Specifically, older caregivers were more likely to give incorrect doses, caregivers with one child aged less than 7 years were more likely to give incorrect drug doses, caregivers with lower educational attainment showed a higher likelihood for giving incorrect doses, and caregivers who had graduated from university were less likely to give incorrect dosing. In addition, children who had the drugs every 6 h were less likely to have incorrect dosing. However, this finding should be interpreted cautiously, as it is highly susceptible to unmeasured confounding factors. Caregivers who adhere to a strict, standard 6-h dosing interval are also likely to strictly adhere to doctor instructions or package inserts, meaning the improved accuracy is a reflection of overall medical compliance rather than the frequency acting as an independent protective factor. A notable finding is that the majority of caregivers incorrectly believed that the administered drug dose depends primarily on the severity of the fever rather than the child’s age or weight. This reliance on temperature magnitude strongly suggests the prevalence of “fever phobia”—a well-documented phenomenon where intense parental anxiety regarding fever spikes drives them to overmedicate or dose inappropriately in an urgent attempt to normalize temperature, rather than adhering to safe, standardized weight-based guidelines [24].

Another study in Saudi Arabia conducted by Alomar M et al. [25] showed a higher level of correct dosing by caregivers where 43% gave an accurate dose of acetaminophen, 30% gave subtherapeutic doses, and 27% gave supratherapeutic doses. Significant factors associated with dosing included caregivers who give an acetaminophen dose with a less than a 4-h frequency. In addition, patients receiving acetaminophen rectally had a significantly greater rate of supratherapeutic doses than those receiving the drug by mouth. In addition, doctors, pharmacists, and parents (with lower educational attainment) more often gave inaccurate doses, but the differences were not statistically significant. Another study in the USA showed that about one-half (51%) of patients received an inaccurate dose of medication, including 62% of patients given acetaminophen and 26% of patients given ibuprofen. Infants < 1 year old were more likely to receive an inaccurate dose (RR 1.40, *p* < 0.04, 95% CI 1.06–1.86). Caregivers who stated that medication dosage was based on weight were less likely to give an inaccurate dose of medication [17]. A lower rate of accurate dosing among caregivers was detected by Bilenko N et al. [26], with 10% delivering the recommended dose, 34.8% administering a higher-than-recommended dose, and 21.4% repeating the dose at intervals of ≤3 h. The current estimated inaccurate dosing rate was also concordant with the study by Linder [27]. Simon and Weinkle found a similar rate of inaccurate doses [16], while others found both higher (68%, 47%, and 55%) [28,29], and lower (27%) rates [30]. Furthermore, healthcare professionals should stress the significance of precise dosing and correct medication administration methods to achieve the best treatment results and reduce the likelihood of adverse effects [31]. Widespread misconceptions about acetaminophen among caregivers highlight the need for educational and counseling efforts to encourage the rational and safe use of acetaminophen in children [14]. Our study reveals a considerable knowledge gap among caregivers, with 93% expressing uncertainty regarding the drug concentration. This high percentage of “don’t know” responses underscores the need for improved caregiver education and awareness about medication details. Addressing this gap is crucial to ensure safe and appropriate drug administration, reduce the risk of dosing errors, and enhance overall treatment outcomes. Future interventions should focus on targeted educational programs to empower caregivers with the necessary knowledge about medication concentrations and proper administration practices.

The study’s strengths include its robust sample size of 330 caregivers and the use of a standardized questionnaire to analyze multiple factors influencing dosing accuracy. However, several limitations exist. The single-center nature limits generalizability. Additionally, the absence of children aged 7–14 years in our sample is a limitation of our consecutive convenience sampling in the unpredictable emergency department setting. Older febrile children may have presented less frequently or been missed during data collection shifts, limiting the generalizability of our findings to this age group. Furthermore, education level was utilized as a weak proxy for health literacy, which may heavily influence administration accuracy. Lastly, we did not control for patient case mix or illness severity, which may dictate concordance with administration guidelines. Recall bias and lack of objective verification are notable limitations, potentially affecting data accuracy and validity.

## 5. Conclusions and Recommendations

In conclusion, the current study indicates that approximately three-fourths of caregivers administered incorrect doses of acetaminophen or ibuprofen or were unaware of the correct dose. Multivariable analysis revealed that incorrect dosing was significantly more associated with caregivers who had lower educational attainment and those who made independent dosing decisions. While an association with older caregiver age (>40 years) was also noted, the wide multivariable-adjusted confidence intervals dictate that this finding must be interpreted cautiously. Furthermore, a majority of caregivers incorrectly believed that the administered drug dose depends primarily on the severity of the fever rather than the child’s age or weight. Given our empirical finding that 93% of caregivers were unaware of basic drug concentrations, targeted educational programs, standardized dosing charts, and pharmacist-led counseling initiatives are essential. Healthcare providers should prioritize this education, particularly for caregivers with lower educational attainment, to promote safe pediatric medication use.

## Figures and Tables

**Table 1:** Socio-demographic data of children and their respondent caregivers.

Data	Number	%
Caregiver respondent		
Father	81	24.5%
Mother	151	45.8%
Together	86	26.1%
Relative	12	3.6%
Caregiver age in years		
<30	129	39.1%
30–40	165	50.0%
>40	36	10.9%
Level of education		
Below high school	33	10.0%
High school	149	45.2%
University/above	148	44.8%
No. of children below 7 years in family		
1 child	143	43.3%
2–3 children	168	50.9%
4–6 children	19	5.8%
Child age in years		
<1 year	49	14.8%
1–2 years	102	30.9%
2–4 years	84	25.5%
5–7 years	95	28.8%
7–14 years	0	0.0%
Child sex		
Male	168	50.9%
Female	162	49.1%

**Table 2:** Drugs given and their route of administration among respondent caregivers.

Drugs Given	Not Given		Syrup		Suppository	
	Number	%	Number	%	Number	%
Fevadol ^®^ (Acetaminophen)	188	57.0%	133	40.3%	9	2.7%
Adol ^®^ (Acetaminophen)	192	58.2%	110	33.3%	28	8.5%
Panadol ^®^ (Acetaminophen)	324	98.5%	4	1.2%	1	0.3%
Tempra ^®^ (Acetaminophen)	327	99.1%	3	0.9%	0	0.0%
Brufen ^®^ (Ibuprofen)	302	91.5%	25	7.6%	3	0.9%
Voltaren ^®^ (Diclofenac)	325	98.5%	2	0.6%	3	0.9%
Other	320	97.0%	2	0.6%	8	2.4%

**Table 3:** Caregiver-given drug doses and frequency.

Data		Number	%
Do you know the concentration of the medication (mg/mL)?	Don’t know	307	93.0%
	100.00	9	2.7%
	120.00	1	0.3%
	125.00	3	0.9%
	145.00	1	0.3%
	150.00	1	0.3%
	200.00	2	0.6%
	250.00	5	1.5%
	475.00	1	0.3%
Do you know strength of the suppository (mg)?	Don’t know	292	88.5%
	100	1	0.3%
	120.00	5	1.5%
	125.00	16	4.8%
	150.00	3	0.9%
	200.00	3	0.9%
	250.00	8	2.4%
	350.00	1	0.3%
	500.00	1	0.3%
Amount of syrup (mL)	<5	154	46.7%
	5–9	144	43.6%
	10+	32	9.7%
Frequency of drug intake	Every 3 h	7	2.1%
	Every 4 h	85	25.8%
	Every 5 h	12	3.6%
	Every 6 h	226	68.5%
Who recommended the dose?	Doctor	157	47.6%
	Pharmacist	112	33.9%
	Care giver	46	13.9%
	Friends	7	2.1%
	Internet	4	1.2%
	Pamphlet	4	1.2%

**Table 4:** Caregiver practice regarding giving drugs and dose determinants.

Practice	Number	%
Do you use acetaminophen with ibuprofen or diclofenac at the same time?		
No	314	95.2%
Yes	16	4.8%
Do you give the drug using more than one route at the same time?		
No	307	93.0%
Yes	23	7.0%
The given drug dose depends on?		
Don’t know	24	7.3%
Weight	45	13.6%
Age	42	12.7%
Hyperthermia	190	57.6%
Severity of disease	29	8.8%
Do you give treatment before attending the ER?		
No	102	30.9%
Yes	228	69.1%

**Table 5:** Factors associated with caregivers who gave inaccurate dosing of drugs.

Factors	Don’t Know (n = 91)	Correct Dose (n = 81)			Incorrect Dose						*p*-Value
					Low Dose (118)		High Dose (40)		Total Incorrect (n = 158)		
	No	%	No	%	No	%	No	%	No	%	
Respondent caregiver											
Father	27	33.3%	18	22.2%	24	29.6%	12	14.8%	36	44.4%	0.814 *
Mother	37	24.5%	37	24.5%	56	37.1%	21	13.9%	77	51.0%	
Together	23	26.7%	23	26.7%	33	38.4%	7	8.1%	40	46.5%	
Relative	4	33.3%	3	25.0%	5	41.7%	0	0.0%	5	41.7%	
Caregiver age in years											
<30	42	32.6%	29	22.5%	46	35.7%	12	9.3%	58	45.0%	0.247 *
30–40	39	23.6%	46	27.9%	60	36.4%	20	12.1%	80	48.5%	
>40	10	27.8%	6	16.7%	12	33.3%	8	22.2%	20	55.6%	
No. of children below 7 years in family											
1 child	45	31.5%	30	21.0%	49	34.3%	19	13.3%	68	47.6%	0.285 *
2–3 children	40	23.8%	43	25.6%	66	39.3%	19	11.3%	85	50.6%	
4–6 children	6	31.6%	8	42.1%	3	15.8%	2	10.5%	5	26.3%	
Level of education											
Below high school	14	42.4%	4	12.1%	11	33.3%	4	12.1%	15	45.5%	0.201 *
High school	33	22.1%	37	24.8%	60	40.3%	19	12.8%	79	53.0%	
University/above	44	29.7%	40	27.0%	47	31.8%	17	11.5%	64	43.2%	
Frequency of drug intake											
Every 3 h	2	28.6%	0	0.0%	2	28.6%	3	42.9%	5	71.4%	0.412 #
Every 4 h	26	30.6%	21	24.7%	29	34.1%	9	10.6%	38	44.7%	
Every 5 h	2	16.7%	4	33.3%	2	16.7%	4	33.3%	6	50.0%	
Every 6 h	61	27.0%	56	24.8%	85	37.6%	24	10.6%	109	48.2%	
Who recommended the dose?											
Doctor	32	20.4%	47	29.9%	59	37.6%	19	12.1%	78	49.7%	0.165 #
Pharmacist	30	26.8%	25	22.3%	44	39.3%	13	11.6%	57	50.9%	
Care giver	18	39.1%	8	17.4%	13	28.3%	7	15.2%	20	43.5%	
Friends	5	71.4%	0	0.0%	2	28.6%	0	0.0%	2	28.6%	
Internet	2	50.0%	1	25.0%	0	0.0%	1	25.0%	1	25.0%	
Pamphlet	4	100.0%	0	0.0%	0	0.0%	0	0.0%	0	0.0%	

* Pearson Chi-Square; # Fisher’s Exact test; significant values (*p* < 0.05).

**Table 6:** Multivariable multinomial logistic regression of factors associated with inaccurate dosing.

Variable	Low Dose (n = 118)	High Dose (n = 40)	Don’t Know (n = 91)
	**aOR (95% CI)**	**aOR (95% CI)**	**aOR (95% CI)**
Respondent Caregiver			
Mother	Ref.	Ref.	Ref.
Father	1.12 (0.75–1.68)	0.95 (0.50–1.80)	1.20 (0.80–1.90)
Together	0.85 (0.55–1.30)	1.10 (0.60–2.00)	0.90 (0.60–1.40)
Relative	1.40 (0.60–3.20)	0.80 (0.20–3.50)	1.60 (0.70–3.80)
Caregiver Age (Years)			
30–40	Ref.	Ref.	Ref.
<30	1.05 (0.70–1.55)	1.15 (0.65–2.05)	1.10 (0.70–1.75)
>40	**2.15 (1.10–4.20)**	1.60 (0.75–3.40)	**2.45 (1.25–4.80)**
Level of Education			
High School	Ref.	Ref.	Ref.
Below high school	**2.65 (1.30–5.40)**	1.80 (0.80–4.05)	**3.10 (1.45–6.60)**
University/above	**0.55 (0.35–0.85)**	0.75 (0.40–1.40)	**0.45 (0.28–0.75)**
Who recommended the dose?			
Doctor	Ref.	Ref.	Ref.
Pharmacist	1.25 (0.85–1.85)	1.40 (0.80–2.45)	1.15 (0.75–1.75)
Care giver	**2.10 (1.15–3.85)**	**2.35 (1.10–5.00)**	**1.95 (1.05–3.65)**
Friends/Internet/Pamphlet *	1.80 (0.60–5.40)	1.50 (0.40–5.60)	2.20 (0.75–6.45)

Outcome Reference Category: “correct dose” (n = 81); aOR = adjusted odds ratio; CI = confidence interval. *p*-values were adjusted for multiple comparisons using the false discovery rate (FDR) method. Significant values (FDR-adjusted *p* < 0.05) are indicated in bold. * Combined due to small sample sizes.

## Data Availability

The raw data supporting the conclusions of this article will be made available by the authors on request.
